# Sigmatropic rearrangements of cyclopropenylcarbinol derivatives. Access to diversely substituted alkylidenecyclopropanes

**DOI:** 10.3762/bjoc.15.29

**Published:** 2019-02-05

**Authors:** Guillaume Ernouf, Jean-Louis Brayer, Christophe Meyer, Janine Cossy

**Affiliations:** 1 Laboratory of Organic Chemistry, Institute of Chemistry, Biology, Innovation (CBI), ESPCI Paris, CNRS (UMR 8231), PSL Research University, 10 rue Vauquelin, 75231 Paris Cedex 05, France; 2 Minakem, Parc Naturel Régional Scarpe-Escaut, 145 chemin des lilas, 59310 Beuvry-la-Forêt, France

**Keywords:** alkylidenecyclopropanes, cyclopropanes, cyclopropenes, sigmatropic rearrangements, strained rings

## Abstract

Cyclopropenes constitute useful precursors of other classes of compounds incorporating a three-membered ring. Although the transformation of substituted cyclopropenes into alkylidenecyclopropanes can be accomplished through different strategies, this review is focusing specifically on the use of [2,3]- and [3,3]-sigmatropic rearrangements involving cyclopropenylcarbinol derivatives as substrates. These sigmatropic rearrangements, which have been developed in recent years, allow a remarkably efficient and stereoselective access to a wide variety of heterosubstituted and/or functionalized alkylidenecyclopropanes which would not be readily accessible by other strategies. The different [2,3]- and [3,3]-sigmatropic rearrangements of cyclopropenylcarbinol derivatives disclosed to date, as well as the analysis of their substrate scope and some applications of the products arising from those reactions, are presented in this review.

## Introduction

**Scheme 1:**
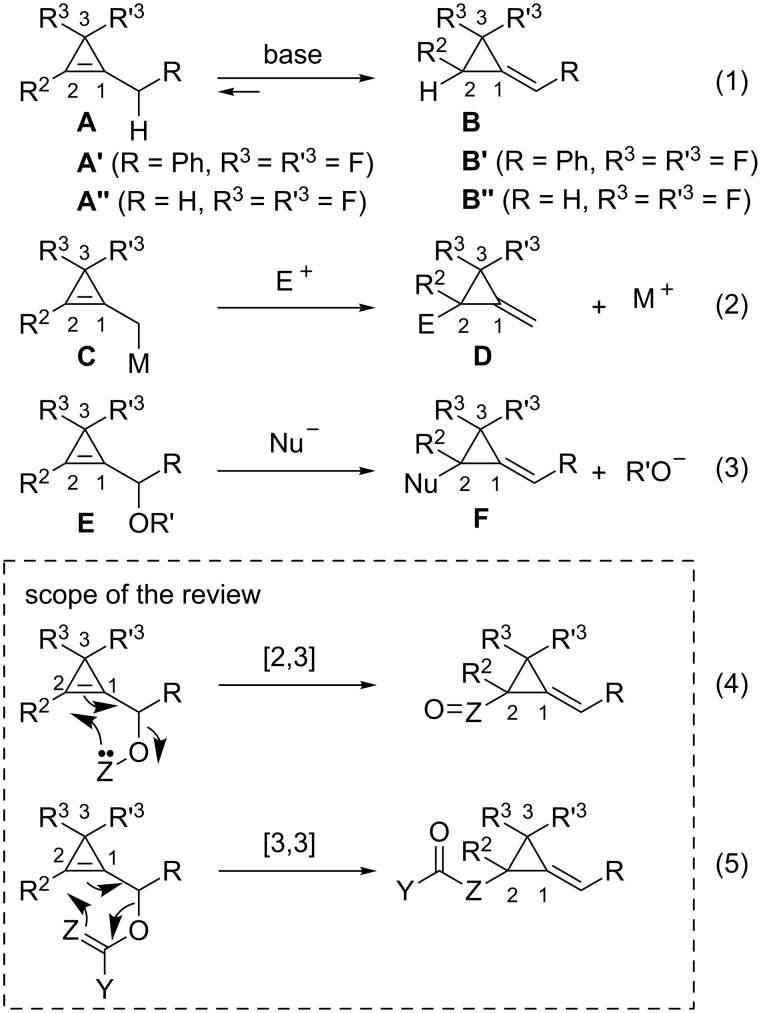
Representative strategies for the formation of alkylidenecyclopropanes from cyclopropenes and scope of the review.

Among the ever expanding diversity of chemical transformations involving cyclopropenes, which are largely dominated by ring-cleavage processes to access functionalized acyclic compounds or to construct new carbocycles or heterocycles, those reactions that preserve the three-membered ring and enable access to diversely substituted cyclopropanes or alkylidenecyclopropanes are also synthetically useful [1–6]. The importance of this latter class of transformations is obviously related to the widespread occurrence of cyclopropanes in natural and/or bioactive compounds [7–8] and the great interest of the cyclopropyl core in new drugs development [9]. Alkylidenecyclopropanes also constitute another important class of strained carbocycles displaying a versatile chemistry owing to their multiple reactive sites (the exocyclic olefin and the proximal and distal bonds on the ring) [10–15]. Although the synthesis of alkylidenecyclopropanes can be achieved by many different routes, controlling the configuration of the exocyclic olefin as well as that of stereocenters on and adjacent to the three-membered ring remains a challenging task [15]. In this context, cyclopropenes can serve as useful precursors of substituted and functionalized alkylidenecyclopropanes. The transformation of cyclopropenes into alkylidenecyclopropanes has been achieved through different strategies (Scheme 1). The first one relies on the isomerization of the olefin in alkylcyclopropenes **A** from the endocyclic to the exocyclic position (Scheme 1, reaction 1) [16–18]. Owing to the relief of ring-strain, the formation of the alkylidenecyclopropane **B** is generally thermodynamically favored [19–20]. However, in the particular case of *gem*-difluorocyclopropenes **A’** (R^3^ = R'^3^ = F) which possess a cyclopropenium (aromatic) character, the position of the equilibrium depends on the substituent at C1. Whereas conjugation with the phenyl group (R = Ph) provides the driving force for the base-promoted isomerization of 1-benzyl-3,3-difluorocyclopropene (**A’**, R = Ph) into the corresponding benzylidene(*gem*-difluoro-cyclopropane) (**B’**) [18], methylene(*gem*-difluorocyclopropane) (**B’’**, R = H) is isomerized into 1-methyl-3,3-difluorocyclopropene (**A”**) [21] (Scheme 1, reaction 1). Another approach relies on the reaction of cyclopropenylmethyl organometallic species **C** with electrophiles through an S_E_2’ process leading to substituted alkylidenecyclopropanes **D** (Scheme 1, reaction 2). Examples of those transformations include the carboxylation of a (trimethylsilylmethyl)cyclopropene in the presence of a fluoride promoter [22], and also the addition of electrophiles to (lithiomethyl)cyclopropenes generated by lithiation of the corresponding methylcyclopropenylsulfone [23] or -sulfoxide [24]. More recently, the addition of cyclopropenylmethylboronates to aldehydes was also reported [25]. A complementary strategy involves the addition of nucleophiles, in particular organometallic reagents, to cyclopropenylcarbinols or their derivatives **E**, which leads to alkylidenecyclopropanes **F** through a formal S_N_2’ process (Scheme 1, reaction 3) [23,26–33]. Thus, methylenecyclopropanes have been prepared by diastereoselective addition of Grignard reagents to cyclopropenylmethyl ethers, possessing a hydroxymethyl directing substituent at C3, in the absence or in the presence of a catalyst (copper or iron salt) [28–30]. Another representative transformation is the copper-catalyzed addition of Grignard reagents to secondary unprotected cyclopropenylcarbinols which proceeds with high levels of chirality transfer to afford alkylidenecyclopropanes possessing a quaternary stereocenter at C2 [31,33]. In this review, we shall exclusively focus on alternative strategies that rely either on a [2,3]-sigmatropic rearrangement (Scheme 1, reaction 4) or a [3,3]-sigmatropic rearrangement of cyclopropenylcarbinol derivatives (Scheme 1, reaction 5). These transformations have emerged as useful tools over the past few years to access hetero-substituted and/or functionalized alkylidenecyclopropanes.

## Review

### [2,3]-Sigmatropic rearrangements involving cyclopropenylcarbinol derivatives

**Scheme 2:**
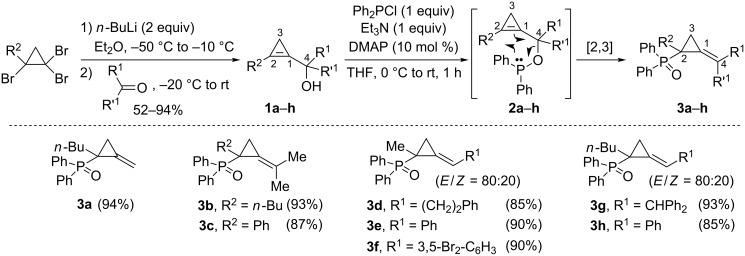
[2,3]-Sigmatropic rearrangement of phosphinites **2a**–**h**.

Following their report on the synthesis of chiral alkylidenecyclopropanes by copper-catalyzed addition of Grignard reagents to enantiomerically enriched cyclopropenylcarbinols [31], Marek et al. investigated other classes of transformations involving those latter strained analogs of allylic alcohols as substrates. In 2007, the [2,3]-sigmatropic rearrangement of cyclopropenylcarbinyl phosphinites was reported as a route to chiral phosphines possessing an alkylidenecyclopropane backbone [34]. The starting cyclopropenylcarbinols were readily prepared by addition of the corresponding cyclopropenyl organolithium reagents, generated in situ by treatment of 1,1,2-tribromocyclopropane with *n*-butyllithium (2 equiv) [35], to various aldehydes and ketones. Marek et al. observed that the treatment of cyclopropenylcarbinols **1a**–**h** with chlorodiphenylphosphine in the presence of triethylamine (THF, rt) resulted in a very rapid formation of (alkylidenecyclopropyl)diphenylphosphine oxides **3a**–**h** (85–94%), resulting from an efficient [2,3]-sigmatropic rearrangement of the in situ-generated phosphinites **2a**–**h**. Primary or tertiary cyclopropenylcarbinols reacted equally well, as shown with the formation of phosphine oxides **3a** (94%), **3b** (93%) and **3c** (87%). The [2,3]-sigmatropic rearrangement of phosphinites **2d**–**h** derived from secondary cyclopropenylcarbinols led to the corresponding phosphine oxides **3d**–**h** (85–93%) as a 80:20 mixture of *E*/*Z* geometric isomers, regardless of the substituent of the alcohol (at C4) and of the cyclopropene (at C2, Scheme 2) [33–34].

**Scheme 3:**
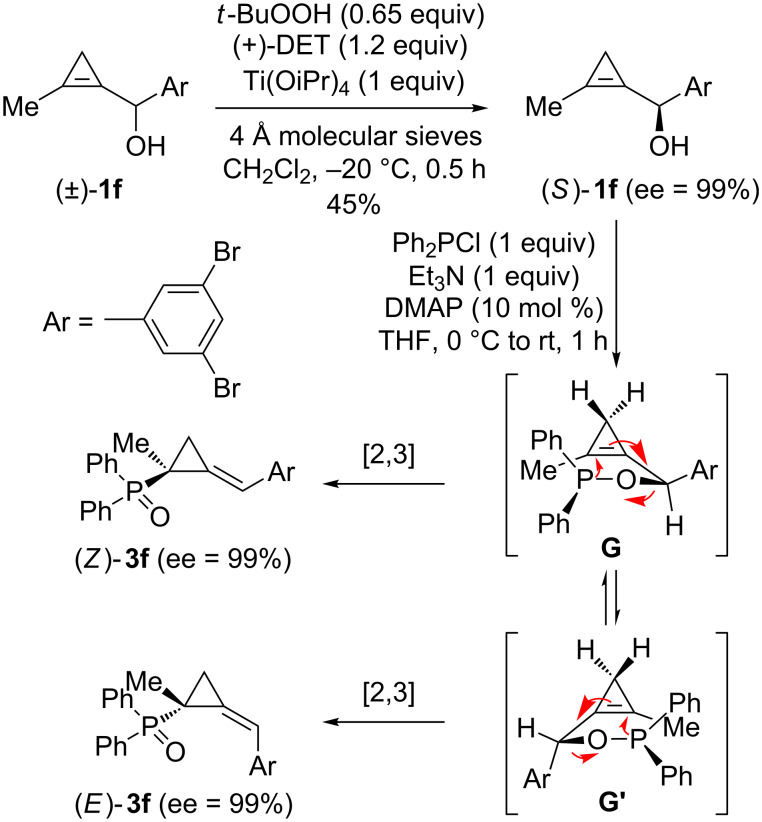
[2,3]-Sigmatropic rearrangement of a phosphinite derived from enantioenriched cyclopropenylcarbinol (*S*)-**1f**.

The [2,3]-sigmatropic rearrangement of an optically enriched phosphinite **2f**, prepared from the corresponding secondary cyclopropenylcarbinol (*S*)-**1f** (ee = 99%), which in turn is readily available by applying the Sharpless kinetic resolution procedure to (±)-**1f** [31], was also investigated. The resulting geometric isomers (*Z*)-**3f** and (*E*)-**3f**, which were separated by flash chromatography, were found to possess optical purities identical to that of the parent substrate (*S*)-**1f** (ee = 99%) thereby confirming that complete chirality transfer occurred (from C4 to C2) during the [2,3]-sigmatropic rearrangement [33–34]. It is also worth mentioning that the absolute configuration of (*Z*)-**3f** and (*E*)-**3f**, which is opposite at C2, was assigned by comparison of their computed and experimentally observed CD spectra [33–34]. To tentatively explain the observed stereochemical outcome in the absence of additional knowledge on the transition state of the rearrangement [36], two reactive conformers **G** and **G’** were considered which would lead to five-membered ring transition states in which the aryl group occupies a preferential pseudo-equatorial or a less favorable pseudo-axial orientation, respectively (Scheme 3) [33–34].

**Scheme 4:**
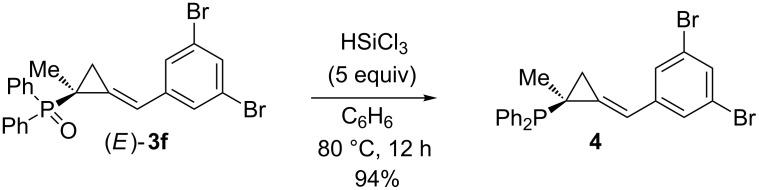
Selective reduction of phosphine oxide (*E*)-**3f**.

The authors also showed that phosphine oxide (*E*)-**3f** could be reduced to the corresponding phosphine **4** (94%) by treatment with trichlorosilane, without affecting the (arylmethylene)cyclopropane moiety (Scheme 4).

**Scheme 5:**
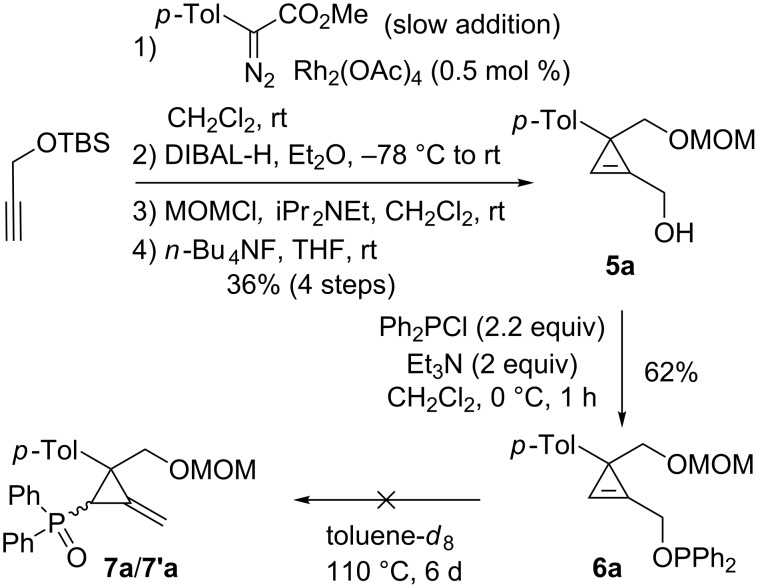
Attempted thermal [2,3]-sigmatropic rearrangement of phosphinite **6a**.

The great efficiency of the [2,3]-sigmatropic rearrangement of phosphinites **2a**–**h** lacking substituents at C3 is in striking contrast with the reactivity of phosphinites possessing a geminal disubstitution at C3. In 2007, Rubin et al. reported their results on the [2,3]-sigmatropic rearrangement of cyclopropenylmethyl phosphinites derived from primary cyclopropenylcarbinols [37]. As illustrated in the case of **5a**, the substrates were prepared from the *tert*-butyldimethylsilyl (TBS) ether of propargyl alcohol by a rhodium-catalyzed cyclopropanation with an aryldiazoacetate followed by reduction of the ester moiety and protecting group manipulation. Phosphinite **6a**, generated from alcohol **5a** under standard conditions, did not undergo a [2,3]-sigmatropic rearrangement into the corresponding diastereomeric phosphine oxides **7a**/**7’a**, even upon prolonged heating (toluene, 110 °C), and underwent slow decomposition instead (Scheme 5).

**Scheme 6:**
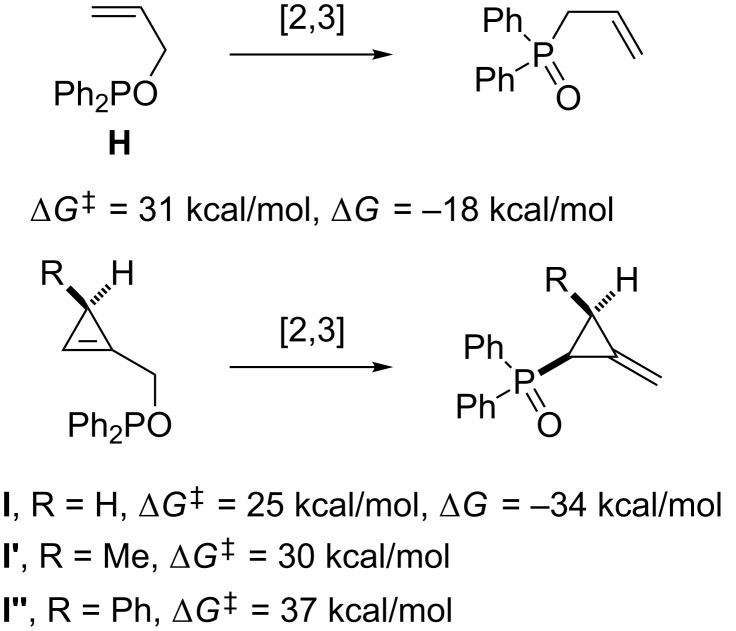
Computed activation barriers and free enthalpies.

This observation was in agreement with DFT calculations which indicated that the rearrangement of cyclopropenylmethyl phosphinite **I**, although thermodynamically favored, displays a high activation barrier compared to that of the acyclic allyl analog **H**. An even higher activation barrier was calculated in the case of the 3-methyl and 3-phenyl-substituted cyclopropenes, **I’** and **I’’**, respectively, which indicates that the concerted [2,3]-sigmatropic rearrangement would require high temperatures incompatible with such thermally labile strained substrates (Scheme 6) [37].

**Scheme 7:**
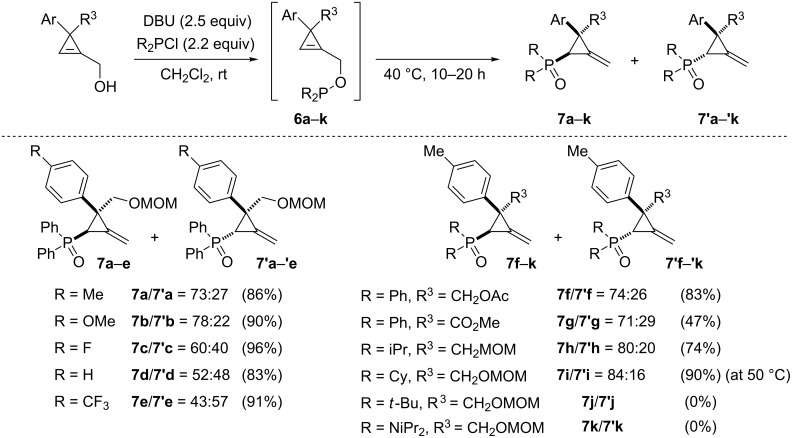
[2,3]-Sigmatropic rearrangement of phosphinites **6a**–**j**.

Interestingly, the authors detected traces of methylenecyclopropanes **7a**/**7’a** when phosphinylation of alcohol **5a** was conducted at room temperature for several hours which led them to consider that the amine could play a role in promoting the [2,3]-sigmatropic rearrangement. After a screening of different tertiary amines, Rubin et al. found that DBU could be used as a base in the phosphinylation reaction but also as an efficient catalyst for the subsequent [2,3]-sigmatropic rearrangement of phosphinite **6a** which afforded a 73:27 mixture of the diastereomeric phosphine oxides **7a**/**7’a** (86%). The major diastereomer **7a** corresponds to a sigmatropic rearrangement occurring on the most hindered face (*cis* to the aromatic group) of the cyclopropene which was somewhat surprising. Substitution at the *para*-position of the aromatic group at C3 significantly affected the diasteomeric ratio with an increase observed with the mesomeric donor methoxy group in favor of diastereomer **7b** (**7b**/**7’b** = 78:22) compared to **7a/7’a**, and a drop of diastereoselectivity when a fluorine atom (**7c**/**7’c** = 60:40) or a hydrogen atom (**7d**/**7’d** = 52:48) were present. An inversion of the face selectivity was detected in favor of diastereomer **7’e** (**7e**/**7’e** = 43:57) arising from the rearrangement of phosphinite **6e** possessing a *p*-trifluoromethylphenyl substituent. Replacement of the acetal protecting group of the hydroxymethyl substituent at C3 (R^3^ = CH_2_OMOM = CH_2_OCH_2_OMe) by an acetate (R^3^ = CH_2_OAc) did not affect the results, as illustrated in the case of **7f**/**7’f**, but the presence of an ester moiety (R^3^ = CO_2_Me) led to the rearranged phosphine oxides **7g**/**7’g** in rather low yield (47%) although the diasteromeric ratio remains similar to that observed for **7a**/**7’a**. Other substituents were tolerated on the phosphorus atom including an isopropyl or a cyclohexyl group and the corresponding phosphine oxides **7h**/**7’h** and **7i**/**7’i** were isolated in good yields. Increasing the steric hindrance around the phosphorus atom resulted in a higher diastereoselectivity. However, the sigmatropic rearrangement of the highly hindered di(*tert-*butyl)phosphinite **6j** and tetra(isopropyl) phosphorodiamidite **6k** did not occur (Scheme 7) [37].

**Scheme 8:**
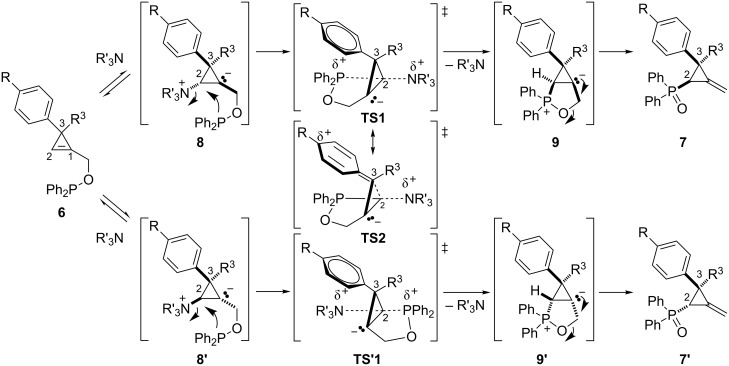
Proposed mechanism for the Lewis base-catalyzed rearrangement of phosphinites **6**.

The mechanism proposed by Rubin et al. involves a reversible addition of the Lewis base (DBU) on the cyclopropene double bond at C2 leading to zwitterronic intermediates **8** and **8’**. This would result in an increase of conformational flexibility thereby facilitating the nucleophilic displacement of the ammonium by the phosphinite through transition states **TS1** and **TS2** (S_N_2-type process), respectively. Oxaphospholanium zwitterions **9** and **9’** would then be obtained and would eventually produce the diastereomeric phosphine oxides **7** and **7’**. Computational studies indicated that the facial selectivity of the initial attack of the Lewis base (DBU) was not responsible for the observed diastereocontrol because of the low difference between the activation barriers of the reactions leading to **8** and **8’**, regardless of the aromatic substituent. Since **8** and **8’** were in rapid equilibrium with phosphinite **6**, the diastereoselectivity should depend on the relative stabilities of the transition states **TS1** and **TS’1**. An electron-donating group at the *para-*position of the aromatic ring could contribute to the stabilization of **TS1**, in which the Ar–C3–C2–P dihedral angle is close to 0°, by considering the mesomeric form **TS2**. The observed dependence of the diastereoselectivity on the σ^+^ Hammett constant of the *para* substituents further supported the proposed mechanism (Scheme 8) [37].

To date and to the best of our knowledge, reports on [2,3]-sigmatropic rearrangements of cyclopropenylcarbinol derivatives appear to be limited to the synthesis of alkylidenecyclopropanes incorporating a phosphorus atom. Cyclopropenylcarbinol derivatives can also lead to other heterosubstituted alkylidenecyclopropanes by using [3,3]-sigmatropic rearrangements.

### [3,3]-Sigmatropic rearrangements involving cyclopropenylcarbinol derivatives

#### Access to heterosubstituted alkylidenecyclopropanes

**Scheme 9:**
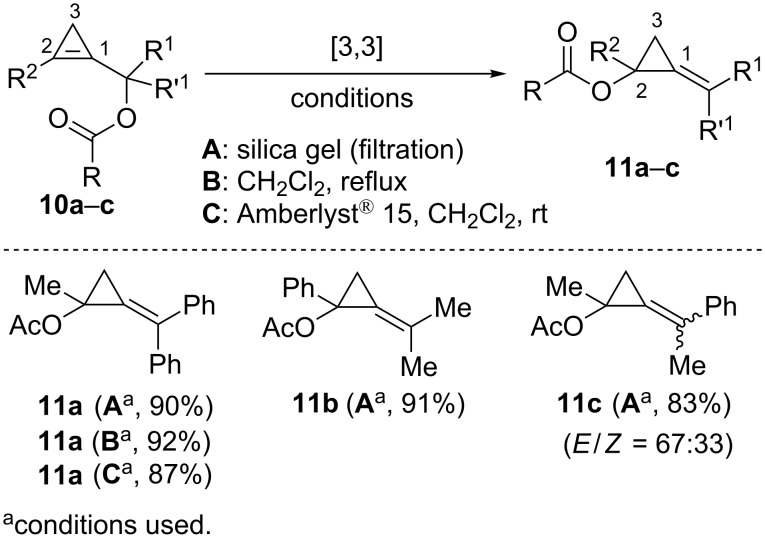
[3,3]-Sigmatropic rearrangement of tertiary cyclopropenylcarbinyl acetates **10a**–**c**.

The interest of secondary cyclopropenylcarbinol derivatives in [3,3]-sigmatropic rearrangements was first highlighted by Marek et al. who investigated the transposition of cyclopropenylcarbinyl esters [33–34]. The [3,3]-sigmatropic rearrangement of acetate **10a** took place during filtration on silica gel and afforded alkylidene(acetoxycyclopropane) **11a** in 90% yield. The ease with which the rearrangement of **10a** occurred was attributed to the relief of ring strain but also to the favorable conjugation of the olefin with the two phenyl groups (R^1^ = R’^1^ = Ph). Alkylidenecyclopropane **11a** could also be obtained in similar yields (92% or 87%, respectively) by heating acetate **10a** in dichloromethane at reflux or by treatment with dry Amberlyst^®^ 15 (a sulfonic acid resin) [33–34]. The rearrangement of the tertiary acetates **10b** (R^1^ = R’^1^ = Me) and **10c** (R^1^ = Ph, R’^1^ = Me) could also be achieved by filtration through silica gel and led to **11b** (91%) and **11c** (83%). The latter non-symmetrical tetrasubstituted alkene **11c** was obtained as a 67:33 mixture of geometric isomers (Scheme 9) [33–34].

**Scheme 10:**
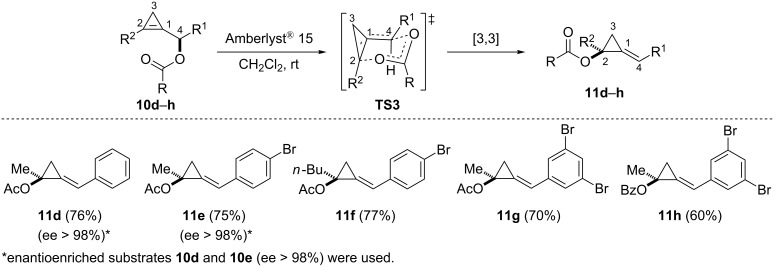
[3,3]-Sigmatropic rearrangement of secondary cyclopropenylcarbinyl esters **10d**–**h**.

The rearrangement of secondary cyclopropenylcarbinyl acetates **10d**–**g** could be achieved in the presence of Amberlyst^®^ 15 and led exclusively to the (*E*)-alkylidene(acetoxycyclopropanes) **11d**–**g** (*E*/*Z* > 99:1) in good yields (70–77%). The acetate could also be replaced by a benzoate as illustrated with the formation of alkylidenecyclopropane **11h** (60%) from substrate **10h**. The authors mentioned that the sigmatropic rearrangement did not proceed under such mild conditions for substrates possessing an alkyl group instead of an aryl group at C4 but no additional details were provided. The high diastereoselectivity was explained by considering a six-membered chair-like cyclic transition state model **TS3** in which the substituent at the α position of the ester (C4) preferentially occupies a pseudo-equatorial position. Although a cationic mechanism could have also been envisioned under the acidic conditions used, the optically enriched acetates **10d** and **10e** (ee > 98%) led to the corresponding alkylidenecyclopropanes **11d** and **11e** with complete chirality transfer (ee > 98%) at C2, thereby probing the concerted suprafacial nature of the rearrangement (Scheme 10) [33–34]. The acidic promotor may be simply assisting the dissociation of the C4–O bond in the transition state **TS3** whilst an aromatic group (R^1^ = Ar) would contribute to the stabilization of a developing positive charge at C4.

The [3,3]-sigmatropic rearrangement of cyclopropenylcarbinyl acetates provides a straightforward and stereoselective entry to alkylidene(acyloxycyclopropanes). Only a few compounds of this family had been previously generated by photochemical reactions (from 4-isopropylidene-3,3-dimethyl-1-thietan-2-thione [38] or from a 4-alkylidene-Δ^1^-pyrazoline [39]) or by pyrolysis of the sodium salt of 3-propionyloxytetramethylcyclobutanone tosyl hydrazone [40]. It is also worth mentioning that completely divergent reactivities have also been reported for cyclopropenylcarbinyl esters in the presence of transition metal catalysts [41–42].

Alkylidene(aminocyclopropane) derivatives constitute another interesting class of heterosubstituted alkylidenecyclopropanes which have been previously synthesized by a Curtius rearrangement of acyl azides derived from alkylidenecyclopropane carboxylic acids [43] or by elimination reactions applied to suitably substituted aminocyclopropane derivatives [44–46].

**Scheme 11:**
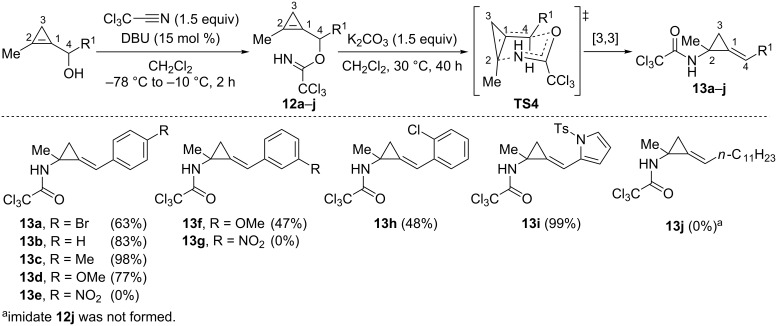
[3,3]-Sigmatropic rearrangement of trichoroacetimidates **12a**–**i**.

In 2014, Hyland et al. disclosed the Overman rearrangement [47] of cyclopropenylcarbinyl trichloroacetimidates [48]. The optimal conditions for the generation of imidates **12a**–**i** involved treatment of secondary cyclopropenylcarbinols with trichloroacetonitrile in the presence of a catalytic amount of DBU (15 mol %) in CH_2_Cl_2_ (−78 °C to −10 °C, 2–3 h) [48–49]. The crude imidates **12a**–**i** were then directly engaged in the [3,3]-sigmatropic rearrangement step which was triggered by heating in the presence of K_2_CO_3_ in CH_2_Cl_2_ (30 °C, 40 h). These latter conditions, which were optimized for imidate **12a**, enabled the formation of *p*-bromobenzylidene[(*N*-trichloroacetylamino)cyclopropane] **13a** as a single (*E*)-isomer in 63% overall yield (two steps from the corresponding alcohol). Compound **13a** was obtained in lower yield in the absence of a base (21%) or when DMF was used as the solvent (53%) though a considerable rate acceleration (22 h instead of 40 h) was observed compared to CH_2_Cl_2_. In the presence of PdCl_2_(MeCN)_2_ (5 mol %), only traces of **13a** were detected and significant decomposition of **12a** took place. As in the case of the [3,3]-sigmatropic rearrangement of cyclopropenylcarbinyl acetates, the observed stereoselectivity was explained by invoking a chair-like transition state model **TS4** in which the aryl group preferentially occupies a pseudo-equatorial orientation (Scheme 11) [48]. Although the presence of a halogen atom was tolerated, as illustrated with the formation of the benzylidenecyclopropanes **13a** (63%) and **13h** (48%), higher yields were obtained in the case of imidates **13b**–**d**, possessing an electro-neutral or an electron-rich aromatic group, which afforded compounds **13b** (83%), **13c** (98%) and **13d** (77%), substituted by a phenyl, a *p*-tolyl or a *p*-anisyl group, respectively. The rearrangement of imidate **12f** possessing a *m*-anisyl substituent afforded benzylidene cyclopropane **13f** in a lower yield (47%) compared to **13d** (77%). The rearrangement of imidate **12i** possessing an electron-rich *N*-tosylpyrrol-2-yl heteroaromatic group, afforded alkylidenecyclopropane **13i** in nearly quantitative yield. Conversely, no rearrangement took place in the case of imidates **12e** and **12g** in which the aromatic group was substituted by a strongly electron-withdrawing nitro group at the *para-* or the *meta-*position, respectively. All these observations point toward the development of a positive charge at the C4 carbon atom (adjacent to the R^1^ substituent) in the transition state **TS4**, as was also suggested previously in the [3,3]-sigmatropic rearrangement of cyclopropenylcarbinyl acetates. Alkylidenecyclopropane **13j** could not be synthesized because trichloroacetimidate **12j** was not obtained by treatment of the corresponding cyclopropenylcarbinol substituted by an *n*-undecyl group with trichloroacetonitrile, even under forcing conditions. The authors tentatively suggested this may be due to the sterically hindered *n*-undecyl chain although this issue was not fully investigated (Scheme 11) [48].

**Scheme 12:**
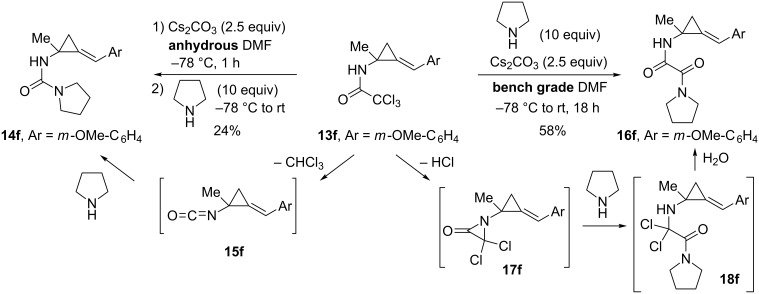
Reaction of trichloroacetamide **13f** with pyrrolidine.

While attempts to access the free alkylidene(aminocyclopropanes) from the corresponding trichloroacetamides proved unsuccessful by hydrolysis (1 M aqueous HCl or KOH, EtOH) or reduction (DIBAL-H or NaBH_4_), Hyland et al. showed that the treatment of (arylmethylene)cyclopropane **13f** with Cs_2_CO_3_ in anhydrous DMF, followed by the addition of an excess of pyrrolidine, produced urea **14f** (24%) [48–49]. The moderate yield of **14f** was attributed to the instability of the in situ*-*generated isocyanate **15f** under the reaction conditions [50]. When trichloroacetamide **13f** was treated with an excess of pyrrolidine and Cs_2_CO_3_ in bench grade (undried) DMF, the reaction followed a different pathway and delivered α-oxoacetamide **16f** (58%) instead of urea **14f** [48–49]. This type of transformation had already been reported [51] and interpreted by a Favorskii-type mechanism, presumably involving the formation of the *gem*-dichloro-α-lactam intermediate **17f** which would undergo ring opening by nucleophilic addition of pyrrolidine followed by hydrolysis of the resulting α,α-dichloro-α-aminoacetamide **18f** (Scheme 12).

**Scheme 13:**
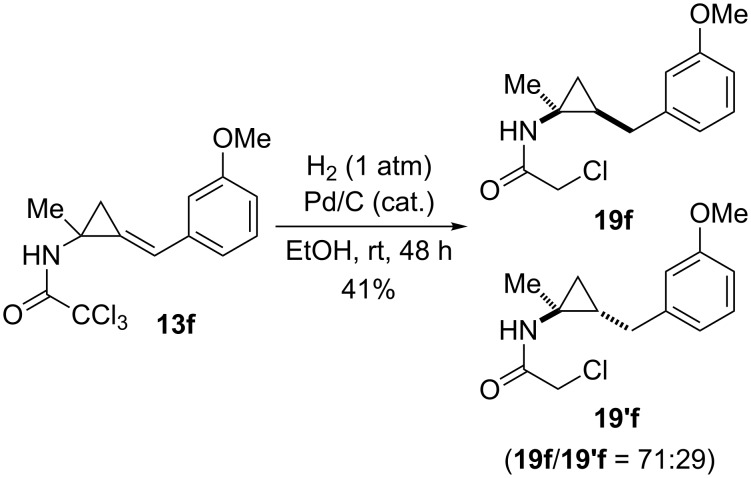
Catalytic hydrogenation of (arylmethylene)cyclopropropane **13f**.

To access aminocyclopropanes, the hydrogenation of (arylmethylene)cyclopropane **13f** was achieved in the presence of Pd/C as a catalyst. Concomitant hydrogenolysis of two carbon–chlorine bonds also took place under these conditions and a 71:29 diastereomeric mixture of the monochloracetamides **19f**/**19’f** was obtained (41%). The rather small difference of steric hindrance between the methyl and the *N*-acylamino group explained the modest face selectivity of hydrogen addition which preferentially occurred on the face of the olefin opposite to the *N-*chloroacetylamino substituent (Scheme 13) [48].

**Scheme 14:**
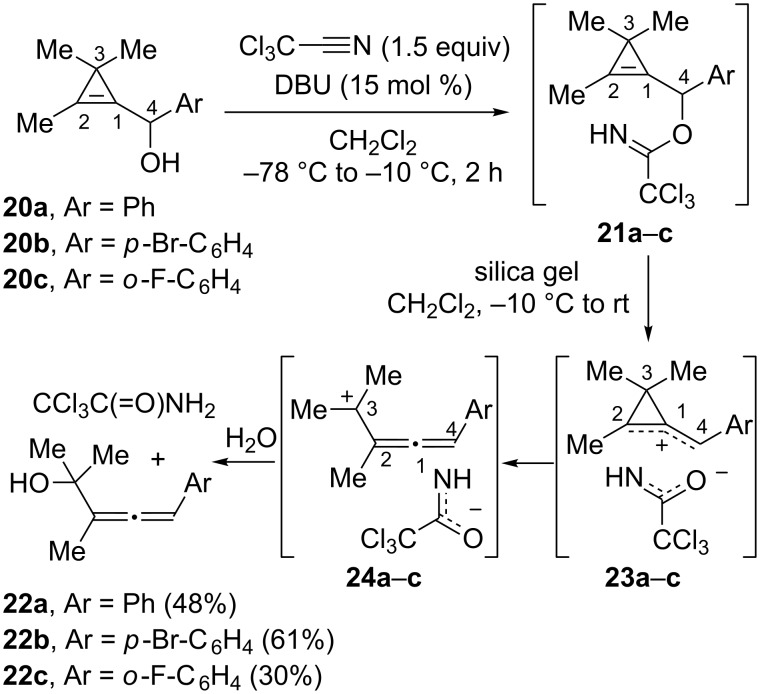
Instability of trichloroacetimidates **21a**–**c** derived from cyclopropenylcarbinols **20a**–**c**.

3,3-Disubstituted cyclopropenylcarbinols could not be used as substrates in the Overman rearrangement. This limitation of the substrate scope is due to the instability of the corresponding trichloroacetimidates. Thus cyclopropenylcarbinols **20a**–**c** possessing *gem*-dimethyl substitution at C3 were converted to imidates **21a**–**c** but upon treatment with silica gel (CH_2_Cl_2_, −10 °C), those latter compounds were converted into α-allenic tertiary alcohols **22a**–**c** (30–61%) The formation of alcohols **22a**–**c** was explained by a mechanism involving ionization of the C4–O bond in imidates **21a**–**c**, followed by ring opening of the alkylidenecyclopropyl cationic intermediates **23a**–**c** [52] and addition of water to the resulting α-allenic carbocations **24a**–**c** (Scheme 14) [48].

**Scheme 15:**
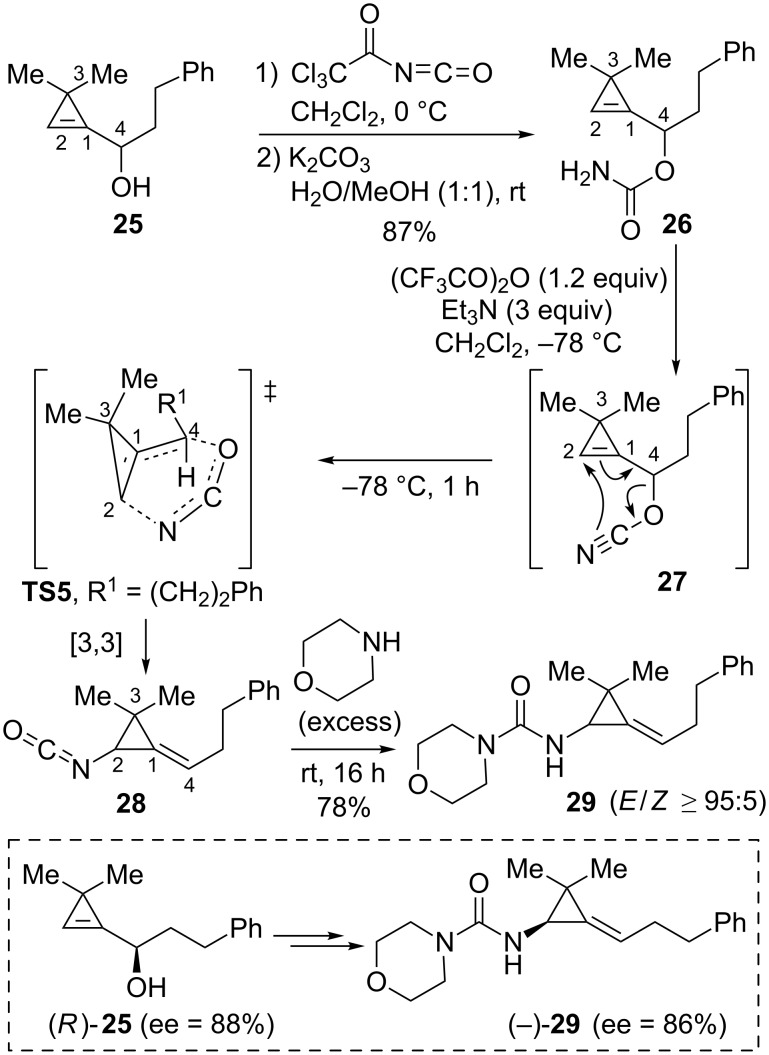
[3,3]-Sigmatropic rearrangement of cyanate **27** generated from cyclopropenylcarbinyl carbamate **26**.

As a complementary strategy, our group examined the [3,3]-sigmatropic rearrangement of cyanates derived from cyclopropenylcarbinols [53]. The allyl cyanate to isocyanate rearrangement displays many interesting features such as the possibility to generate the reactive species by dehydration of carbamates under mild conditions and the ultimate formation of isocyanates which can be derivatized in situ [54]. The conditions were optimized with alcohol **25** substituted by a 2-phenylethyl group at the oxygen-bearing carbon atom (C4) and possessing *gem*-disubstitution at C3 on the three-membered ring. Alcohol **25** was readily converted to carbamate **26** by reaction with trichloroacetyl isocyanate followed by cleavage of the trichloroacetyl group by alkaline hydrolysis. Dehydration of carbamate **26** was achieved by treatment with trifluoroacetic anhydride in the presence of triethylamine under mild conditions (CH_2_Cl_2_, −78 °C) [55] and the in situ-generated cyanate **27** underwent a sigmatropic rearrangement into the corresponding isocyanate **28**. The formation of this reactive isocyanate intermediate was ascertained by the addition of morpholine which enabled the isolation of urea **29** in good yield (78%). It is worth noting that alkylidenecyclopropane **29** was formed with high diastereoselectivity (*E*/*Z* ≥ 95:5) at low temperature (−78 °C) but a slight erosion of diastereoselectivity was observed (*E*/*Z* = 88:12) when the same sequence was performed at 0 °C. The stereochemical outcome was in agreement with a six-membered transition state model **TS5** in which the three atoms of the cyanate (O=C=N) moiety would be arranged in an almost linear fashion (an angle of 173° was calculated in the allyl cyanate to isocyanate transition state) [56] and the substituent at C4 would preferentially occupy a pseudo-equatorial orientation. Additionally, the same sequence applied to the enantioenriched alcohol (*R*)-**25** (ee = 88%) delivered urea (−)-**29** with essentially the same optical purity (ee = 86%), thereby indicating that chirality transfer (from C4 to C2) occurred during the sigmatropic rearrangement of cyanate **27** into isocyanate **28** (Scheme 15) [53].

**Scheme 16:**
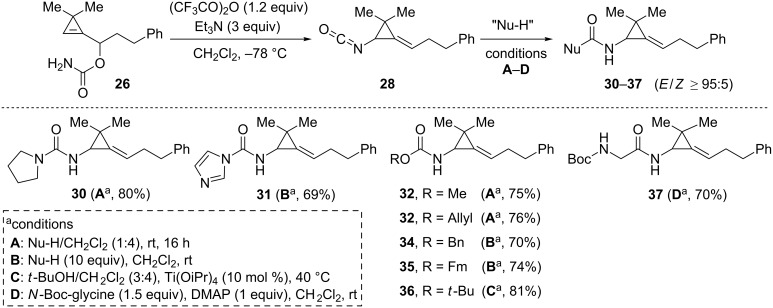
Synthesis of alkylidene(aminocyclopropane) derivatives **30**–**37** from carbamate **26**.

All attempts to isolate isocyanate **28** were unsuccessful but derivatization of this latter reactive intermediate could be achieved in situ by addition of a broad range of nucleophiles, which were either used as co-solvents or added in excess. Thus, reaction with pyrrolidine, imidazole, methanol, allyl alcohol, benzyl alcohol and 9-fluorenemethanol (FmOH) provided the corresponding urea **30**, *N*-carbamoyl imidazole **31** and carbamates **32**–**35**, respectively, in good yields (69–80%). The reaction of isocyanate **28** with *tert*-butanol was sluggish even by heating at 40 °C but could be accelerated by addition of Ti(OiPr)_4_ (10 mol %) to deliver the corresponding *N*-Boc- carbamate **36** (81%). The condensation of isocyanate **28** with *N*-Boc-glycine in the presence of DMAP (Goldschmidt–Wick coupling) [57] provided amide **37** in 70% yield (Scheme 16) [53].

**Scheme 17:**
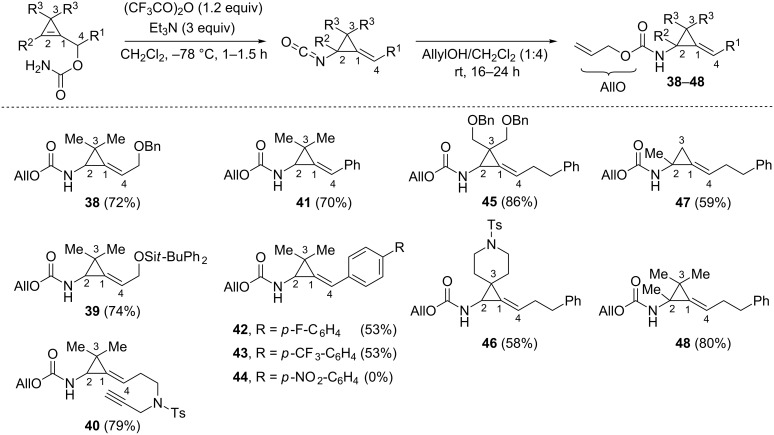
Scope of the dehydration–[3,3]-sigmatropic rearrangement sequence of cyclopropenylcarbinyl carbamates.

The examination of the substrate scope indicated that a broad range of alkyl chains, possibly incorporating heteroatoms, were compatible with the dehydration–[3,3]-sigmatropic sequence, as illustrated with the isolation of compounds **38**–**40** (72–79%) after nucleophilic trapping of the generated isocyanate intermediates with allyl alcohol. Benzylidenecyclopropane **41** was also obtained in good yield (70%) but the efficiency of the sigmatropic rearrangement dropped for carbamates in which the aromatic group at C4 is substituted by an electron-withdrawing group at the *para*-position. Indeed, *N-*Alloc (arylmethylene)(aminocyclopropanes) **42** and **43**, substituted by a *p*-fluorophenyl and a *p*-(trifluoromethyl)phenyl group, respectively, were isolated in moderate yield (53%). Moreover, (*p*-nitrophenylmethylene)cyclopropane **44** could not be obtained under these conditions [53]. These results indicate that the [3,3]-sigmatropic rearrangement of cyclopropenylcarbinyl cyanates, as previously reported for their allylic counterparts [56], does not involve a synchronous process because dissociation of the C4–O bond is more advanced in the transition state **TS5** than the formation of the C2–N bond (Scheme 15). The rearrangement of cyclopropenylcarbinyl cyanates accommodates various substituents at C3, as well as the presence of a substituent at C2 or even a fully substituted cyclopropene ring, as shown with the successful formation of alkylidenecyclopropanes **45**–**48** (58–86%, Scheme 17) [53].

**Scheme 18:**
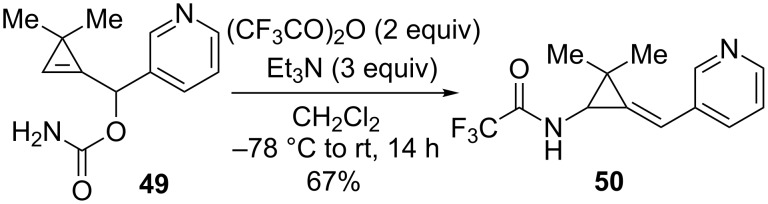
Formation of trifluoroacetamide **50** from carbamate **49**.

Interestingly, alkylidene(isocyanatocyclopropanes) arising from the [3,3]-sigmatropic rearrangement of cyclopropenylcarbinyl cyanates could also be derivatized into trifluoroacetamides. This transformation was discovered fortuitously when carbamate **49** was treated with an excess of trifluoroacetic anhydride (2 equiv) in the presence of Et_3_N (3 equiv) to achieve the dehydration–sigmatropic rearrangement sequence. Trifluoroacetamide **50** (67%) was the product directly formed under these conditions and the Lewis basic character of the pyridine ring was suspected to be responsible for the observed reactivity (Scheme 18).

**Scheme 19:**
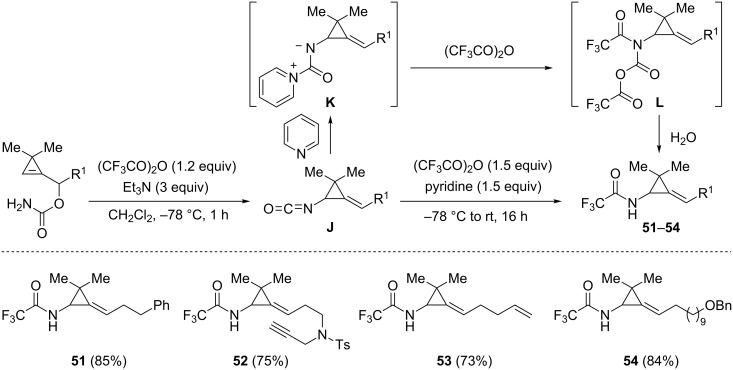
Formation of alkylidene[(*N*-trifluoroacetylamino)cyclopropanes] **51**–**54**.

With the aim of achieving the same derivatization in the case of other substrates devoid of a pyridine ring, several 3,3-dimethylcyclopropenylcarbinyl carbamates were engaged in the dehydration–[3,3]-sigmatropic rearrangement sequence under the previously used conditions but trifluoroacetic anhydride (1.5 equiv) and pyridine (1.5 equiv) were then subsequently added to the reaction mixture. Under these conditions, the corresponding trifluoroacetamides **51**–**54** could be effectively isolated in good yields (73–85%). The addition of pyridine to the isocyanates **J** arising from the [3,3]-sigmatropic rearrangement would probably generate the zwitterionic intermediates **K** which would then react with trifluoroacetic anhydride to produce *N*,*O*-bis(trifluoroacetyl)carbamates **L**. Trifluoroacetamides **51**–**54** would be generated from adducts **L** after hydrolysis of the reaction mixture (Scheme 19) [53].

To control the diastereoselectivity of the hydrogenation of alkylidene[(*N*-acylamino)cyclopropanes] possessing a single substituent at C2, it is possible to rely either on the steric hindrance or on the coordinating ability of the amide group. Thus, the hydrogenation of trifluoroacetamide **51** catalyzed by Pd/C afforded *N*-trifluoroacetylaminocyclopropane **55** as the major diastereomer (**55**/**55’** = 92:8) because of the preferential addition of hydrogen on the less hindered face of the trisubstituted alkene opposite to the trifluoroacetamide moiety. A reversal of face selectivity can be observed by performing a directed iridium(I)-catalyzed hydrogenation in the presence of Crabtree’s catalyst [58] which afforded aminocyclopropane **55’** as the major diastereomer (**55’**/**55** = 90:10, Scheme 20) [53].

**Scheme 20:**
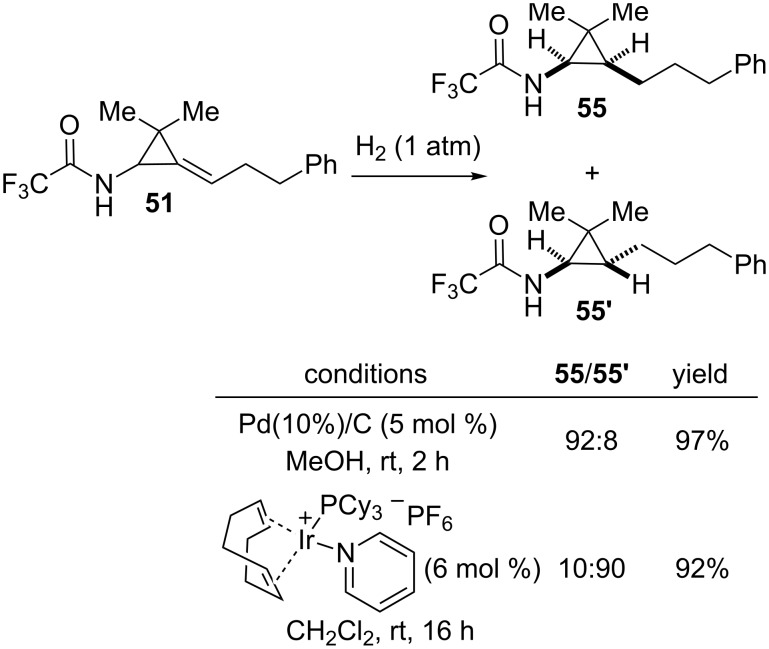
Diastereoselective hydrogenation of alkylidenecyclopropane **51**.

The potential of [3,3]-sigmatropic rearrangements involving cyclopropenylcarbinol derivatives is not restricted to the synthesis of heterosubstituted alkylidenecyclopropanes and was also exploited to access functionalized alkylidenecyclopropanes, with creation of a new carbon–carbon bond on the three-membered ring with the control of two contiguous stereocenters.

#### Ireland–Claisen rearrangement of cyclopropenylcarbinyl esters

**Scheme 21:**
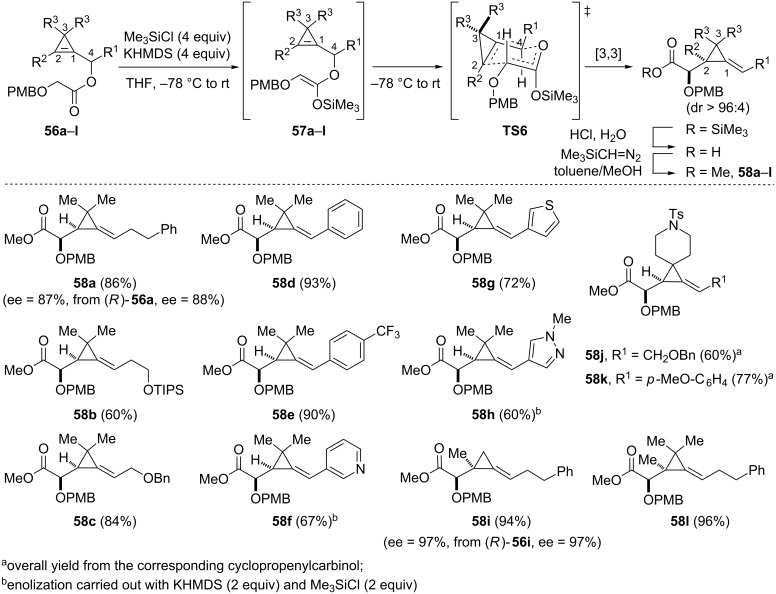
Ireland–Claisen rearrangement of cyclopropenylcarbinyl glycolates **56a**–**l**.

The Ireland–Claisen rearrangement of silyl ketene acetals generated from allylic (or propargylic) esters is arguably one of the most useful variant of the Claisen rearrangement that has found countless applications in organic synthesis [59]. The feasibility of the Ireland–Claisen rearrangement of cyclopropenylcarbinyl esters was investigated in the case of glycolates **56a**–**l** which were readily prepared by coupling of the corresponding cyclopropenylcarbinols with (4-methoxybenzyloxy)acetic acid. Enolization of glycolates **56a**–**l** was carried out by treatment with Me_3_SiCl (4 equiv) followed by addition of KHMDS (usually 4 equiv) in THF at −78 °C. The resulting silyl ketene acetals of (*Z*)-configuration **57a**–**l**, arising from O-silylation of the corresponding chelated potassium enolates [60], underwent an efficient [3,3]-sigmatropic rearrangement upon warming to room temperature. After an acidic work-up and treatment of the crude carboxylic acids with trimethylsilyldiazomethane, the resulting α-alkoxy methyl esters **58a**–**l**, incorporating an alkylidenecyclopropane moiety, were obtained as single detectable diastereomers [61]. As in the previously discussed [3,3]-sigmatropic rearrangements, the observed stereochemical outcome was in agreement with a six-membered chair-like transition state model **TS6** in which the substituent at the α-position of the oxygen atom (C4) preferentially occupies a pseudo-equatorial position. The scope of the reaction is rather broad as the substituent at C4 can be an alkyl chain, possibly incorporating a protected alcohol, as illustrated with the formation of alkylidenecyclopropanes **58a** (86%), **58b** (60%) and **58c** (84%). It is worth mentioning that despite the use of a strong base (KHMDS) and the acidity of the “vinylic” protons of cyclopropenes which is comparable to that of a terminal alkyne [62], cyclopropenylcarbinyl glycolates devoid of substituents at C2 were viable substrates. The sequence allowed access to benzylidenecyclopropane **58d** (93%) and to (arylmethylene)cyclopropane **58e** in excellent yield (90%), despite the presence of the electron-withdrawing trifluoromethyl substituent at the *para*-position of the aromatic ring. Some heteroaromatic groups were also tolerated at C4, as shown with the synthesis of (heteroarylmethylene) cyclopropanes **58f**–**h** (60–72%). The *gem*-dimethyl substitution at C3 which was common to the previous cyclopropenylcarbinyl glycolates **56a**–**h**, could be suppressed and the corresponding alkylidenecyclopropane **58i** was produced in excellent yield (94%). More sterically hindered substituents were tolerated at C3, as illustrated with the isolation of the spirocyclic compounds **58j** (60%) and **58k** (77%), and alkylidenecyclopropane **58l** possessing a fully substituted three-membered ring was also formed in excellent yield (96%). That the Ireland–Claisen rearrangement of cyclopropenylcarbinyl glycolates proceeded with chirality transfer was also verified in the case of alkylidenecyclopropanes **58a** and **58i** which were obtained with optical purities (ee = 87% and ee = 97%, respectively) identical to those of the corresponding enantioenriched precursors (*R*)-**56a** and (*R*)-**56i** (Scheme 21) [61].

**Scheme 22:**
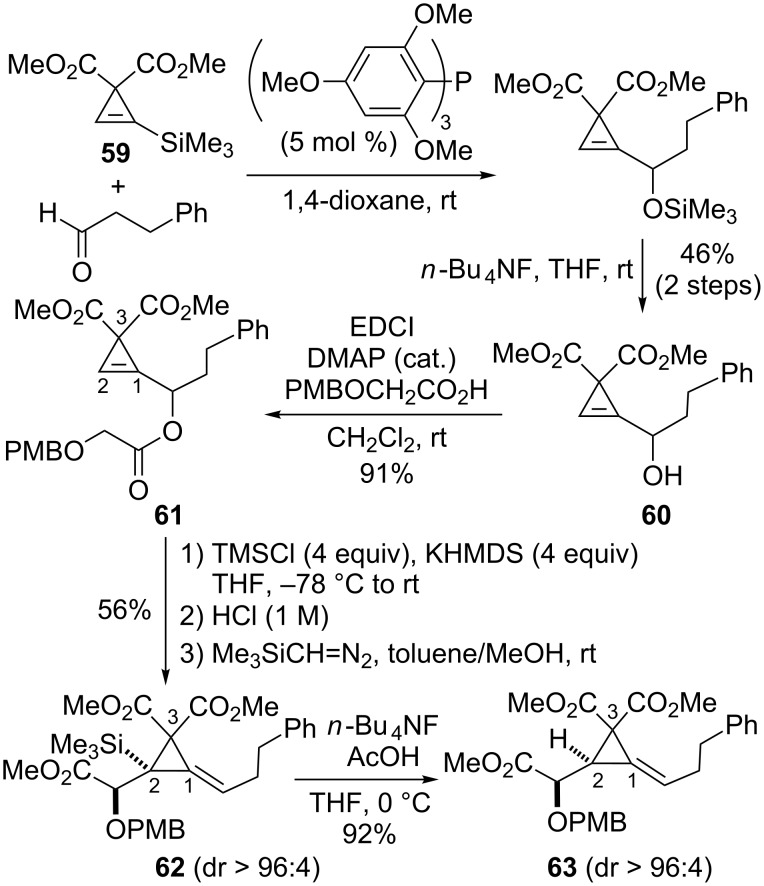
Synthesis and Ireland–Claisen rearrangement of glycolate **61** possessing *gem*-diester substitution at C3.

The addition of a cyclopropenyllithium to an aldehyde is arguably the most widely used method to access cyclopropenylcarbinols but Gevorgyan et al. disclosed an interesting organocatalytic route to cyclopropenylcarbinols possessing *gem*-diester substitution at C3 [63]. As illustrated with the preparation of alcohol **60**, the strategy relies on a sila-Morita–Baylis–Hillman reaction between cyclopropenylsilane **59** and 3-phenylpropanal catalyzed by electron-rich tris(2,4,6-trimethoxyphenyl)phosphine (TTMPP) [63]. After desilylation, cyclopropenylcarbinol **60** was converted into glycolate **61** under standard conditions and the latter ester was engaged in the Ireland–Claisen rearrangement. Because the *gem*-diester substitution at C3 increased the acidity of the proton at C2 in substrate **61** [64], silylation of that position took place under the reaction conditions prior to the Ireland–Claisen rearrangement which eventually produced alkylidenecyclopropane **62** (56%) with high diastereoselectivity. The trimethylsilyl substituent at C2 could then be easily removed by treatment of **62** with tetrabutylammonium fluoride under buffered conditions (AcOH, THF, 0 °C) to afford alkylidenecyclopropane **63** (92%, Scheme 22) [61].

**Scheme 23:**
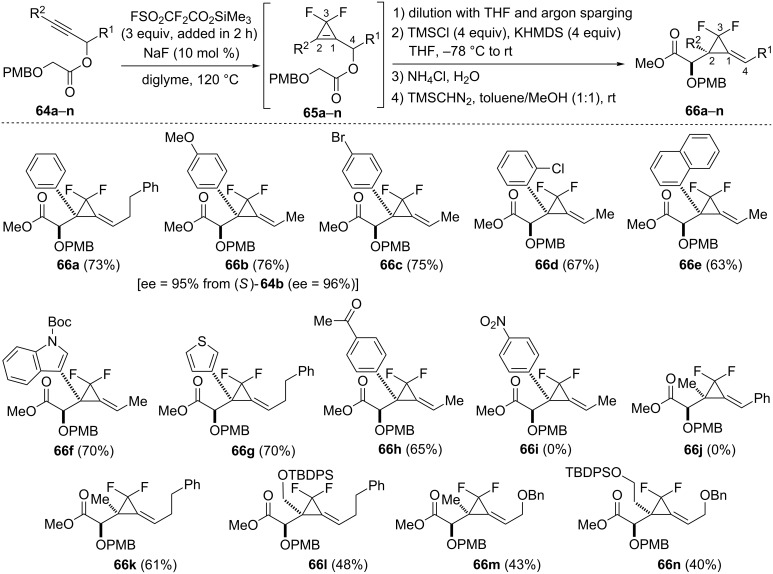
Synthesis of alkylidene(*gem*-difluorocyclopropanes) **66a**–**h**, and **66k**–**n** from propargyl glycolates **64a**–**n**.

The Ireland–Claisen rearrangement was then extended to a challenging class of cyclopropylcarbinyl glycolates possessing *gem*-difluoro substitution at C3 [65]. *Gem*-difluorocyclopropenes are accessible by difluorocyclopropenation of alkynes with difluorocarbene but these compounds display poor stability in most cases and readily undergo hydrolysis into cyclopropenones which possess an aromatic character [66–67]. *Gem*-difluorocyclopropenylcarbinyl glycolates **65a**–**n** were prepared by slow addition of an excess of trimethylsilyl fluorosulfonyldifluoroacetate (TFDA) [68] to a solution of propargyl glycolates **64a**–**n** containing NaF in diglyme at 120 °C. Difluorocyclopropene **65a** could be purified by flash chromatography on silica gel and was isolated in 86% yield but this compound rapidly underwent decomposition upon storage. The instability of glycolates **65a**–**n** was a critical issue which was solved by carrying those intermediate compounds directly in the sigmatropic rearrangement. Byproducts arising from the difluorocyclopropenation reaction (CO_2_, SO_2_ and Me_3_SiF) were simply removed by argon sparging of the reaction mixture and the Ireland–Claisen rearrangement was then triggered by addition of Me_3_SiCl (4 equiv) and KHMDS (4 equiv), (THF, −78 °C to rt). Subsequent hydrolysis and treatment with trimethylsilyldiazomethane afforded the corresponding α-alkoxy methyl esters **66a**–**h**, and **66k**–**n** possessing a 3,3-difluoroalkylidenecyclopropane scaffold. This two-step difluorocyclopropenation–Ireland–Claisen rearrangement sequence was applied to propargyl glycolates **64a**–**e** possessing a phenyl, a *p*-methoxyphenyl, a *p*-bromophenyl, an *o*-chlorophenyl or a 1-naphthyl substituent at the acetylenic position, as illustrated with the formation of compounds **66a**–**e** (63–76%, two steps from the corresponding propargylic glycolates). Not surprisingly, chirality transfer (from C4 to C2) also occurred in the Ireland–Claisen rearrangement, as demonstrated by the formation of (−)-**66b** (ee = 95%) from optically enriched (*S*)-**64b** (ee = 96%). Heteroaromatic groups (indol-3-yl and 3-thienyl) were tolerated at the acetylenic position and the corresponding glycolates **64f** and **64g** led to compounds **66f** and **66g** in 70% yield. A *p*-acetylphenyl group was compatible as shown with the isolation of alkylidenecyclopropane **66h** (65%) but it should be noted that the electron-withdrawing methyl ketone was converted to a trimethylsilyl enol ether upon treatment with KHMDS/Me_3_SiCl. By contrast, an electron-withdrawing *p*-nitrophenyl group was not tolerated because the intermediate cyclopropene **65i** underwent decomposition under the reaction conditions of the Ireland–Claisen rearrangement, presumably because of competitive deprotonation at C4. A phenyl substituent was incompatible at C4 as the corresponding substrate **65j** decomposed upon treatment with KHMDS/Me_3_SiCl. This was explained by a competitive abstraction of the hydrogen at C4 by the base thereby resulting in side reactions. However, various alkyl substituents could be present at the propargylic position in glycolates **64k**–**n** which afforded the corresponding rearranged compounds **66k**–**n** in moderate yields (40–61%, Scheme 23) [65].

**Scheme 24:**
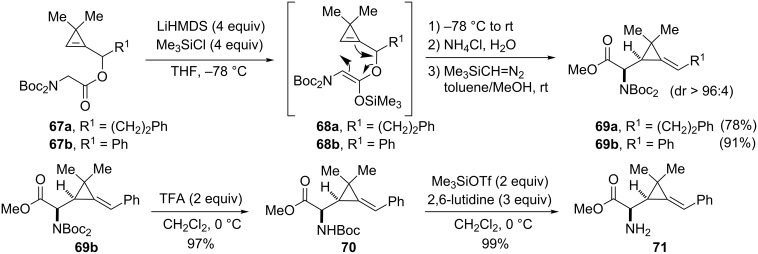
Ireland–Claisen rearrangement of *N*,*N*-diBoc glycinates **67a** and **67b**.

With the goal of accessing α-amino acid derivatives incorporating an alkylidenecyclopropane, the Ireland–Claisen rearrangement of *N*,*N*-diBoc glycinates **67a** and **67b** was explored. The reaction conditions were essentially the same as those described previously with glycolates **56a**–**l** except that LiHMDS was used as the base in the enolization step [69]. The (*Z*)-silyl ketene acetals **68a** and **68b** were generated, in agreement with previous results disclosed by Carbery et al. with allylic *N*,*N*-diBoc glycinates [69], and underwent a Ireland–Claisen rearrangement to afford *N*,*N*-diBoc α-amino esters **69a** (78%) and **69b** (91%) in good yields and with high diastereoselectivity [61]. Although cleavage of the two Boc groups could not be achieved cleanly upon exposure of **69b** to a large excess of trifluoroacetic acid, this operation could be accomplished in a sequential manner by addition of trifluoroacetic acid (2 equiv, CH_2_Cl_2_, 0 °C) and then by treatment of the resulting *N*-Boc carbamate **70** (97%) with trimethylsilyl triflate in the presence of 2,6-lutidine to generate α-amino ester **71** (99%, Scheme 24) [61].

Alkylidenecyclopropanes resulting from the Ireland–Claisen rearrangement of cyclopropenylcarbinyl glycolates and glycinates can serve as useful precursors of other classes of functionalized cyclopropanes. As shown previously with alkylidene(aminocyclopropane) derivatives, diastereoselective hydrogenation reactions of alkylidenecyclopropanes possessing a single substituent at C2 can be carried out with complementary face selectivities, depending on the conditions and substrates. Thus, the hydrogenation of **58a** catalyzed by Rh/C occurred on the less hindered face of the alkene and gave rise to cyclopropyl α-alkoxy ester **72** as a single detectable diastereomer. When Pd/C was used as the catalyst, cleavage of the PMB group took place concomitantly and the α-hydroxy ester **73** arising from addition of hydrogen on the less-hindered face of the olefin was obtained predominantly (**73**/**73’** = 90:10) albeit with lower diastereocontrol compared to the protected alcohol **58a**. Cleavage of the PMB ether in **58a** was achieved purposely with DDQ so that a hydroxy-directed hydrogenation of the resulting α-hydroxy ester **74** could be carried out in the presence of Crabtree’s catalyst [58], thereby allowing access to cyclopropylcarbinol **73’** with high diastereoselectivity (**73’**/**73** = 97:3, Scheme 25).

**Scheme 25:**
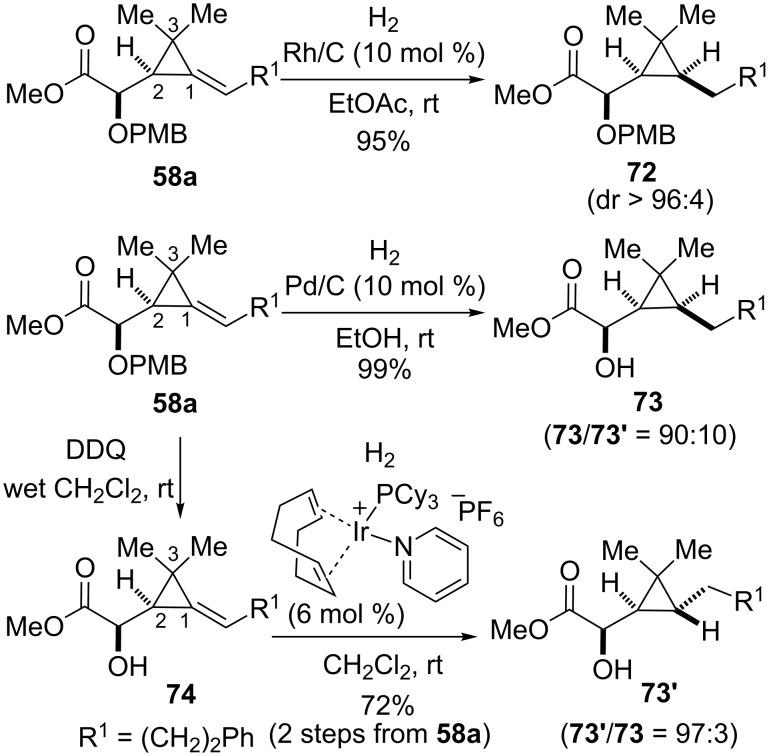
Diastereoselective hydrogenation of alkylidenecyclopropanes **58a** and **74**.

**Scheme 26:**
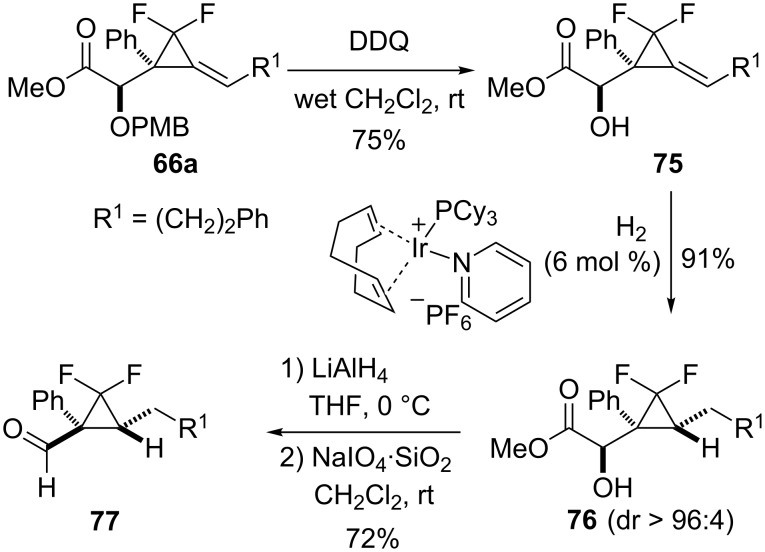
Synthesis of functionalized *gem*-difluorocyclopropanes **76** and **77** from alkylidenecyclopropane **66a**.

By taking advantage of the directing effect of a hydroxy group, diastereoselective hydrogenations of alkylidenecyclopropanes possessing two substituents at C2 could be achieved. As illustrated for alkylidene(*gem*-difluorocyclopropane) **66a**, cleavage of the PMB group and subsequent hydrogenation of the resulting α-hydroxy ester **75** (75%) in the presence of Crabtree’s catalyst delivered the *gem*-difluorocyclopropane **76** (91%) as a single diastereomer. The reduction of ester **76** with LiAlH_4_ and oxidative cleavage of the resulting 1,2-diol with NaIO_4_ delivered the highly substituted *gem*-difluorocyclopropanecarboxaldehyde **77** (72%) possessing a quaternary stereocenter (Scheme 26) [65].

**Scheme 27:**
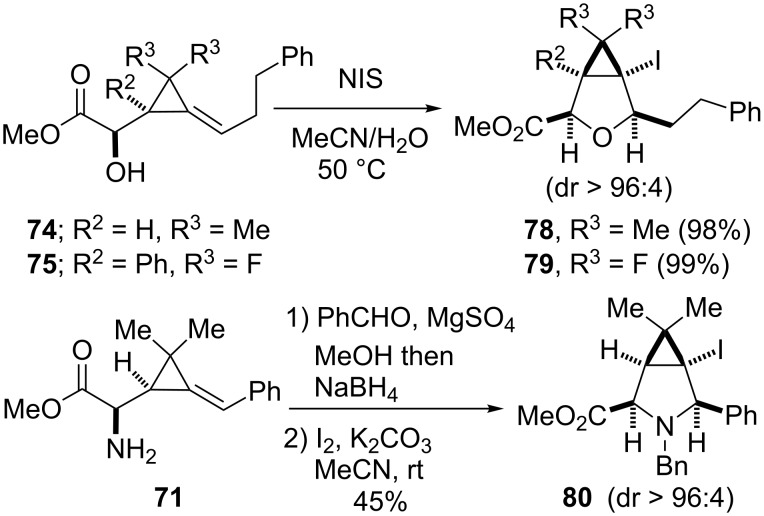
Access to oxa- and azabicyclic compounds **78**–**80**.

Other examples of post-functionalization involve iodolactonization reactions which were applied to α-hydroxy esters **74** and **75** using *N*-iodosuccinimide (MeCN/H_2_O, 50 °C) [70], or to the *N*-benzylamine generated from α-amino ester **71** (by reductive animation with benzaldehyde) in the presence of I_2_ and K_2_CO_3_ (MeCN, rt) [71]. These iodocyclizations led to the oxabicyclic compounds **78** (98%) and **79** (99%), and to the azabicyclic product **80** (45%), respectively, with high diastereoselectivities (Scheme 27) [61,65].

## Conclusion

In recent years, [2,3]- and [3,3]-sigmatropic rearrangements of cyclopropenylcarbinol derivatives have emerged as useful tools for the stereoselective synthesis of a wide variety of alkylidenecyclopropanes, substituted by heteroatoms (P, O, N, F) and/or incorporating valuable functional groups (α-alkoxy or α-amino acid derivatives) which are potentially useful for further functionalization. The reactivity of heterosubstituted/functionalized alkylidenecyclopropanes arising from those sigmatropic rearrangements, which are not easily accessible by other strategies, has only been sparingly investigated to date but the results summarized in this short review, in conjunction with the very rich chemistry of alkylidenecyclopropanes, may stimulate further investigations in this particular area.
